# Large chondroid syringoma in the reconstructed nasal tip: Diagnostic dilemmas and surgical management

**DOI:** 10.1016/j.jdcr.2021.07.014

**Published:** 2021-07-24

**Authors:** Roeland W.H. Smits, Vera van Dis, Frank R. Datema

**Affiliations:** a Department of Otolaryngology and Head and Neck Surgery, Erasmus University Medical Center, Rotterdam, The Netherlands; b Department of Pathology, Erasmus University Medical Center, Rotterdam, The Netherlands

**Keywords:** cartilaginous proliferations, chondroid syringoma, forehead flap, rhinoplasty

## Introduction

Chondroid syringomas of the nose are rarely encountered. We present a novel case of a patient who developed a chondroid syringoma of the nose after paramedian forehead flap reconstruction. Furthermore, we discuss complete excision by external rhinoplasty approach.

## Case report

**Fig 1:**
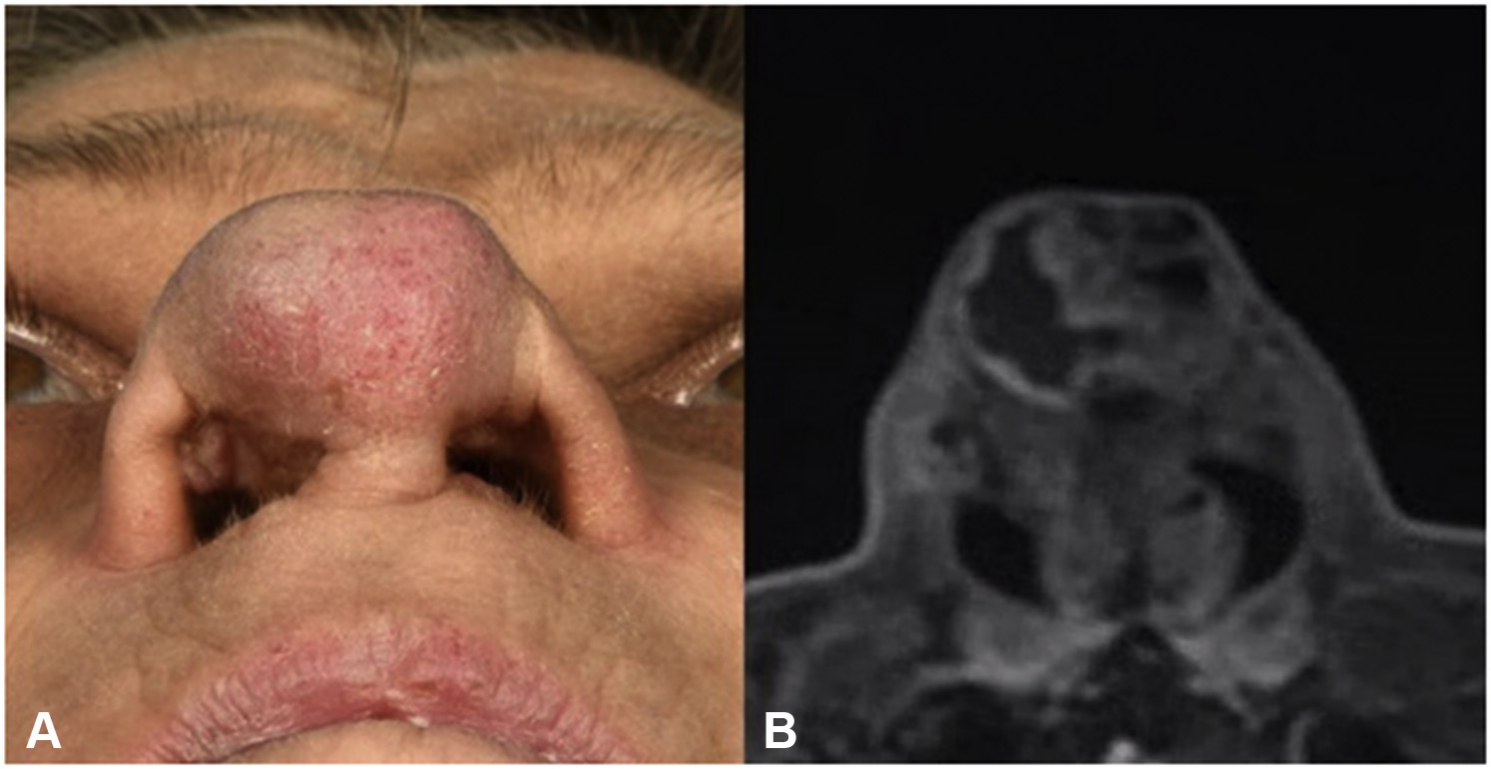
**A,** Basal view of the nasal tumor (*left*) and **(B)** the corresponding T1 MRI sequence (*right*). *MRI*, Magnetic resonance imaging.

A 52-year-old Caucasian woman with a medical history of frontonasal dysplasia and several nasal reconstructive procedures in childhood presented with a slowly progressive, painless swelling of the nasal tip. Symptoms started spontaneously, approximately 4 months earlier. Besides blue discoloration of the skin and a progressive nasal obstruction, there were no other symptoms. Physical examination revealed a subcutaneous deformity of the nasal tip, expanding into the right nasal vestibule (Fig 1, *A*). Scars due to a childhood surgery, including paramedian forehead flap transposition, were noticeable, with no apparent direct relation with the swelling. Fine needle aspiration cytology reported the presence of well–differentiated, cartilaginous proliferation, but no definite diagnosis could be made. Magnetic resonance imaging showed a homogenous tumor (T1: hypointense; T2: hyperintense) of 17 × 17 × 13 mm^3^ with irregular borders, not infiltrating deeper structures (Fig 1, *B*).

**Fig 2:**
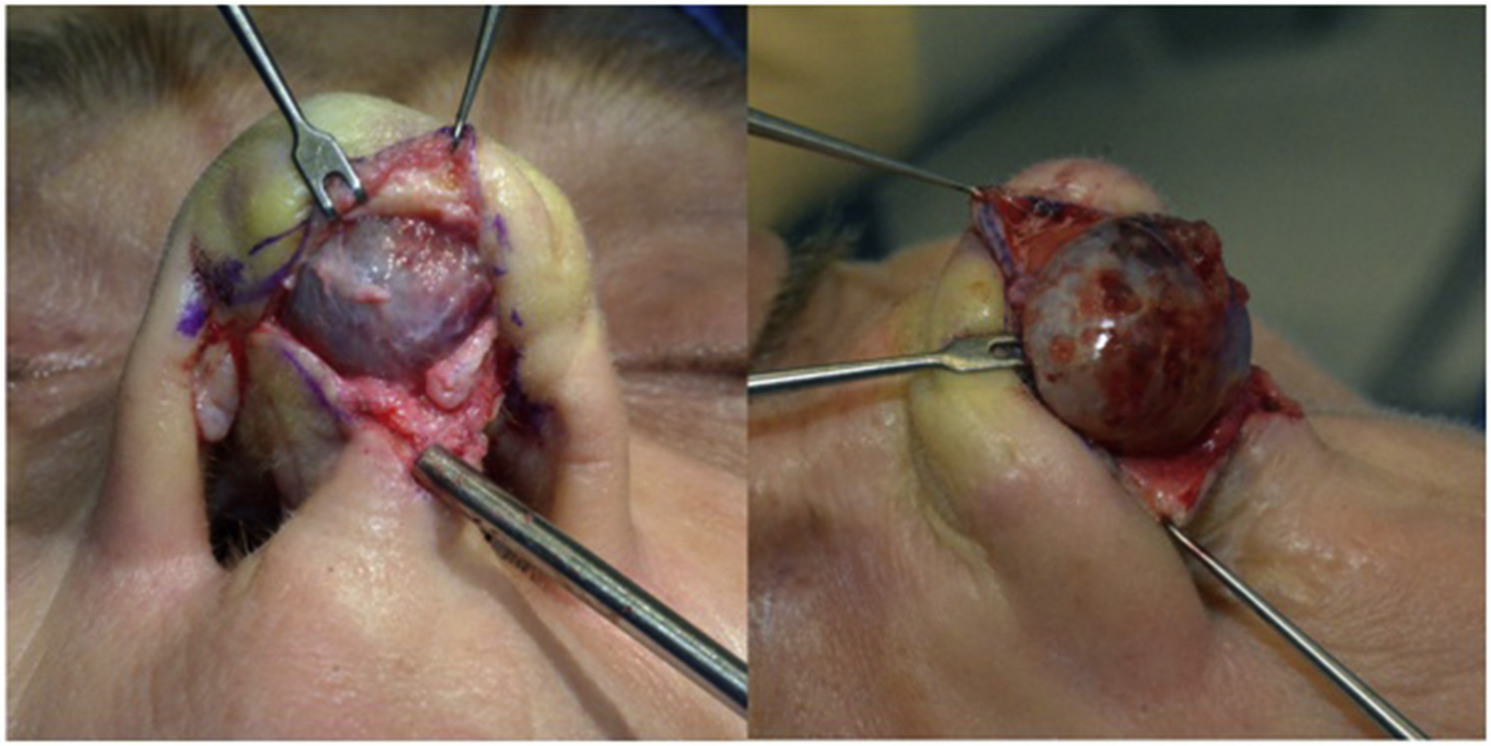
External rhinoplasty approach presenting the tumor.

To avoid visible scar formation at the nasal tip, without compromising tumor exposure, an external rhinoplasty approach was used. An inverted-V, midcolumellar incision, connected to marginal incisions, allowed meticulous, blunt elevation of the skin's soft tissue envelope from a fairly thick, bluish tumor capsule (Fig 2).

**Fig 3:**
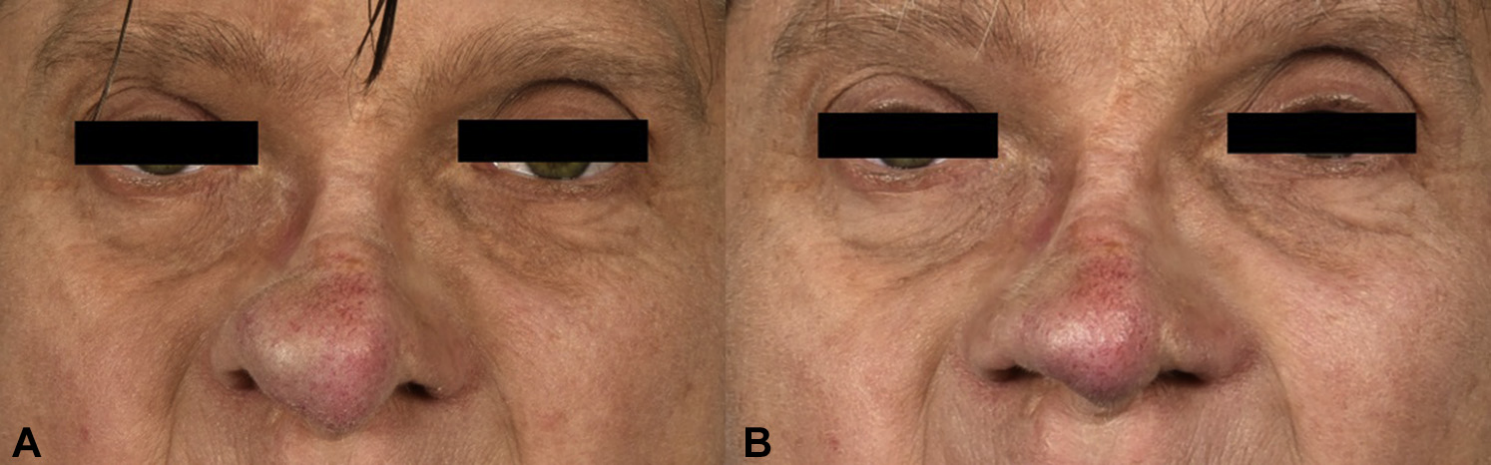
**A,** Appearance of the nose at first presentation (*left*) and **(B)** appearance 10 months after surgery (*right*).

The underlying nasal tip cartilage was slightly eroded. Pending a final diagnosis, the reconstruction of the cartilage and restoration of the tip projection caused by tumor volume loss was left for secondary surgery. Three months following the surgery, the patient was very satisfied with the obtained cosmetic and functional aspects of the nose and was not motivated for secondary rhinoplasty (Fig 3).

**Fig 4:**
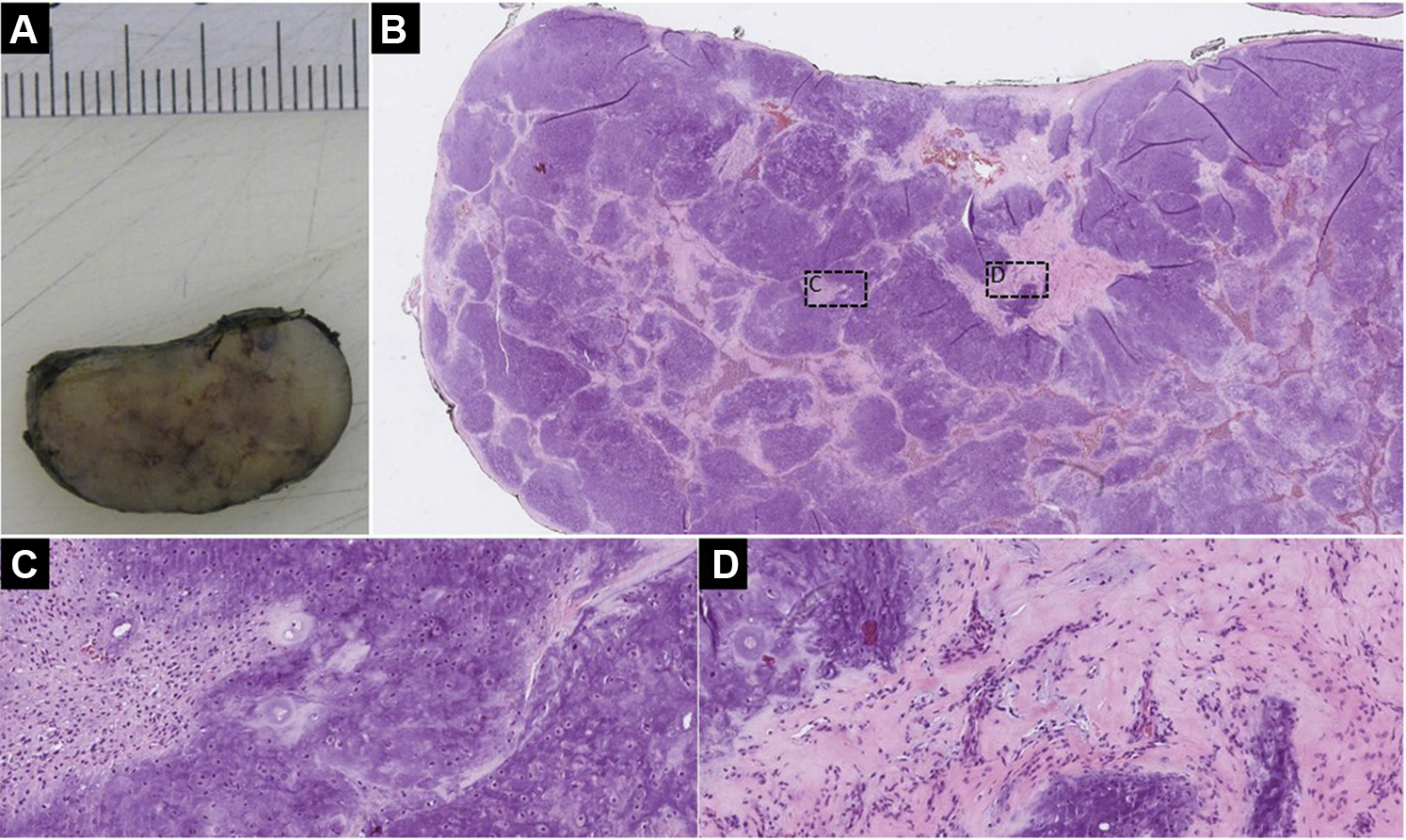
**A,** Cut surface showing a *tan-white* glassy lesion. **B,** In the overview, we see a well-demarcated, encapsulated lesion with a nodular aspect (hematoxylin-eosin stain; original magnification: ×1.25.) **C,** The nodules have a chondroid appearance (hematoxylin-eosin stain; original magnification: ×20), **(D)** with bland, single epithelioid to spindle cells in the surrounding stroma, stained positive for pankeratin (hematoxylin-eosin stain; original magnification: ×20.)

Histology revealed a dermal-based lesion composed almost entirely of hyaline nodules (Fig 4). However, in the surrounding matrix, solitary spindled and epithelioid cells with small nuclei and stained for pankeratin were identified, consistent with a diagnosis of chondroid syringoma or benign mixed tumor.

## Discussion

The term chondroid syringoma was introduced by Hirsch and Helwig^1^ in 1961. An incidence of <0.01% of primary skin tumors makes it a rare diagnosis. Characteristically, these benign skin tumors of 0.5-3.0 cm in the head and neck area of adult men are slow growing and painless.^2^ Their silent presentation and rare occurrence make clinical differentiation difficult for most physicians. The treatment of choice is complete margin-free excision to prevent recurrence. The tumor's histopathologic features constitute a mix of epithelial and mesenchymal cell types with both eccrine and apocrine origins (sweat gland features in a cartilage-like stroma). Fine needle aspiration cytology usually remains ineffective for smaller lesions.^3^ There is no consensus on the role of radiologic examination, but in our patient, we felt that it was important to exclude the possibility of a vascular or infiltrating tumor prior to surgical exploration.

Small chondroid syringomas in the nose have been previously described, with varying (atypical) clinical presentations, including 1 malignant counterpart extending into the paranasal sinus.^4^, ^5^, ^6^, ^7^ The size and clinical features in our patient were similar to a case of chondroid syringoma of the forehead described by Khan et al.^8^ Because a paramedian forehead flap was used to reconstruct the nasal tip in our patient, it is possible that our patient's tumor occurred at this location secondary to the transposition of the skin and soft tissue from the forehead to the nose.

Especially in a patient with a history of extensive reconstructive nasal surgery, it is important to choose a surgical approach that avoids additional visible scaring without compromising tumor exposure and radical dissection. The external rhinoplasty approach meets these criteria and should be considered as an alternative to direct (vertical) transcutaneous tumor exploration.

## Conflicts of interest

None disclosed.
